# Climate change and the emerging ecology of helminthiases: a One Health perspective integrating microbial and environmental drivers

**DOI:** 10.1128/msphere.00265-26

**Published:** 2026-05-29

**Authors:** Lucas Fukui-Silva, Josué de Moraes

**Affiliations:** 1 Research Center on Neglected Diseases, Guarulhos University 92928, Guarulhos, São Paulo, Brazil; 2 Research Center on Neglected Diseases, Scientific and Technological Institute, Brazil University 92928 , São Paulo, Brazil; University of Georgia, Athens, Georgia, USA

**Keywords:** helminthiases, climate change, One Health, microbial ecology, environmental transmission

## Abstract

Helminthiases affect more than one billion people worldwide and remain tightly linked to environmental conditions, yet they are often underrepresented in discussions of climate-sensitive infectious diseases. As global temperatures rise and ecosystems shift, the life cycles, geographic distributions, and transmission dynamics of parasitic helminths are being reshaped in complex and sometimes contrasting ways. Here, we argue that helminthiases should be understood as ecological outcomes emerging from interactions among climatic drivers, environmental conditions, microbial communities, and host populations, rather than as the result of isolated environmental shifts. Drawing on examples from schistosomiasis, soil-transmitted helminthiases, and angiostrongyliasis, as well as climate-sensitive helminths affecting animal populations, we examine how climate change can alter parasite development, host ecology, and environmental persistence. We further highlight the role of microbial communities as mediators of transmission. Finally, we discuss how integrating environmental monitoring, microbiological data, and predictive modeling within a One Health framework can support more adaptive and anticipatory surveillance and control strategies.

## PERSPECTIVE

## HELMINTHIASES IN A WARMING AND ECOLOGICALLY UNSTABLE WORLD

Climate change is transforming the global landscape of infectious diseases (1), yet parasitic helminths remain underrepresented in discussions of climate-sensitive pathogens (2). Their life cycles depend on environmental conditions, ecological interactions, and the availability of intermediate and definitive hosts. Because many developmental stages occur outside the host, helminths are highly sensitive to climatic fluctuations and ecological disturbances. Small shifts in temperature, humidity, hydrology, or vegetation can alter larval survival, accelerate or delay development, and change geographic suitability for host and parasite populations (3).

These sensitivities position helminths as indicators of ecological instability (4, 5). Their distributions are already shifting in response to warming temperatures, altered precipitation patterns, and changing land-use practices. Understanding these transitions requires a framework that integrates climatic, ecological, microbial, and host-related determinants within a One Health perspective.

This environmental dependence is particularly evident in soil-transmitted helminths, including *Ascaris lumbricoides*, *Trichuris trichiura*, and hookworms, whose transmission relies on persistent environmental stages influenced by soil moisture, temperature, and sanitation conditions (6, 7). Such patterns highlight how environmental variability shapes transmission dynamics across different helminth systems. These observations underscore the need for integrative frameworks capable of capturing interactions across environmental, biological, and ecological domains. Such an approach recognizes that human, animal, and environmental health are interconnected and that helminth transmission emerges from the interactions among these domains (8).

In this Perspective, we outline key mechanisms through which climate change reshapes helminth ecology and transmission dynamics. We incorporate recent evidence from schistosomiasis, soil-transmitted helminths, emerging zoonoses such as angiostrongyliasis, and climate-sensitive helminths affecting animal populations to illustrate how climate-driven environmental change influences parasite development, host ecology, and transmission patterns. By doing so, we aim to provide a more mechanistic and integrative framework for understanding helminth emergence in a rapidly changing world.

## INTEGRATING CLIMATE, MICROBIAL ECOLOGY, AND HOST DYNAMICS: A ONE HEALTH PERSPECTIVE

Helminth transmission reflects the combined influence of environmental conditions, microbial communities, and host populations (9). Climate change acts on each of these components, often simultaneously, creating complex and sometimes unexpected patterns of infection. A One Health approach is essential for understanding these dynamics because helminths cross ecological boundaries and depend on interactions among multiple species and habitats (10).

### Climate as a determinant of host ecology

Temperature, precipitation, and extreme weather events strongly influence the distribution and behavior of intermediate and definitive hosts. For example, recent large-scale analyses have shown that climate variability and urbanization are reshaping the spatial distribution of *Schistosoma mansoni* snail hosts, including expansion into peri-urban environments (9). Warming temperatures may shorten developmental times of parasite stages and enable hosts to expand into new regions. Conversely, extreme heat or habitat loss may suppress native host populations while favoring invasive or synanthropic species capable of sustaining transmission. Together, these ecological shifts can create new pathways for helminths to persist or emerge (2).

### Microbial ecology as a modulator of parasite development

Environmental and host-associated microbial communities influence egg viability, larval development, and parasite survival (2). Experimental and observational studies suggest that microbial composition can influence host susceptibility and parasite establishment, particularly in snail–parasite systems where microbiota may modulate infection compatibility (11). These interactions indicate that microbial communities may act as ecological mediators linking environmental change to parasite development and transmission dynamics.

### Anthropogenic environmental change as an amplifier

Deforestation, agricultural expansion, urbanization, and water management practices interact with climatic pressures to reshape habitats, modify soil and water conditions, and alter wildlife and domestic animal movements. These disruptions often concentrate competent hosts or create new ecological niches favorable for parasite persistence. Urban heat islands, for example, can improve survival of certain synanthropic hosts while simultaneously altering microbial communities in soil and water (12). The cumulative impact of climate stress and human-driven disturbance frequently intensifies helminth transmission, particularly in regions with high social vulnerability.

### A conceptual One Health model for helminth transmission

An integrated conceptual model brings together environmental conditions, climatic forces, microbial ecology, and host dynamics (13). Importantly, these interactions are not linear. Climate-driven changes may enhance transmission in some settings while reducing it in others, depending on ecological thresholds and species-specific biology (14).

**Fig 1:**
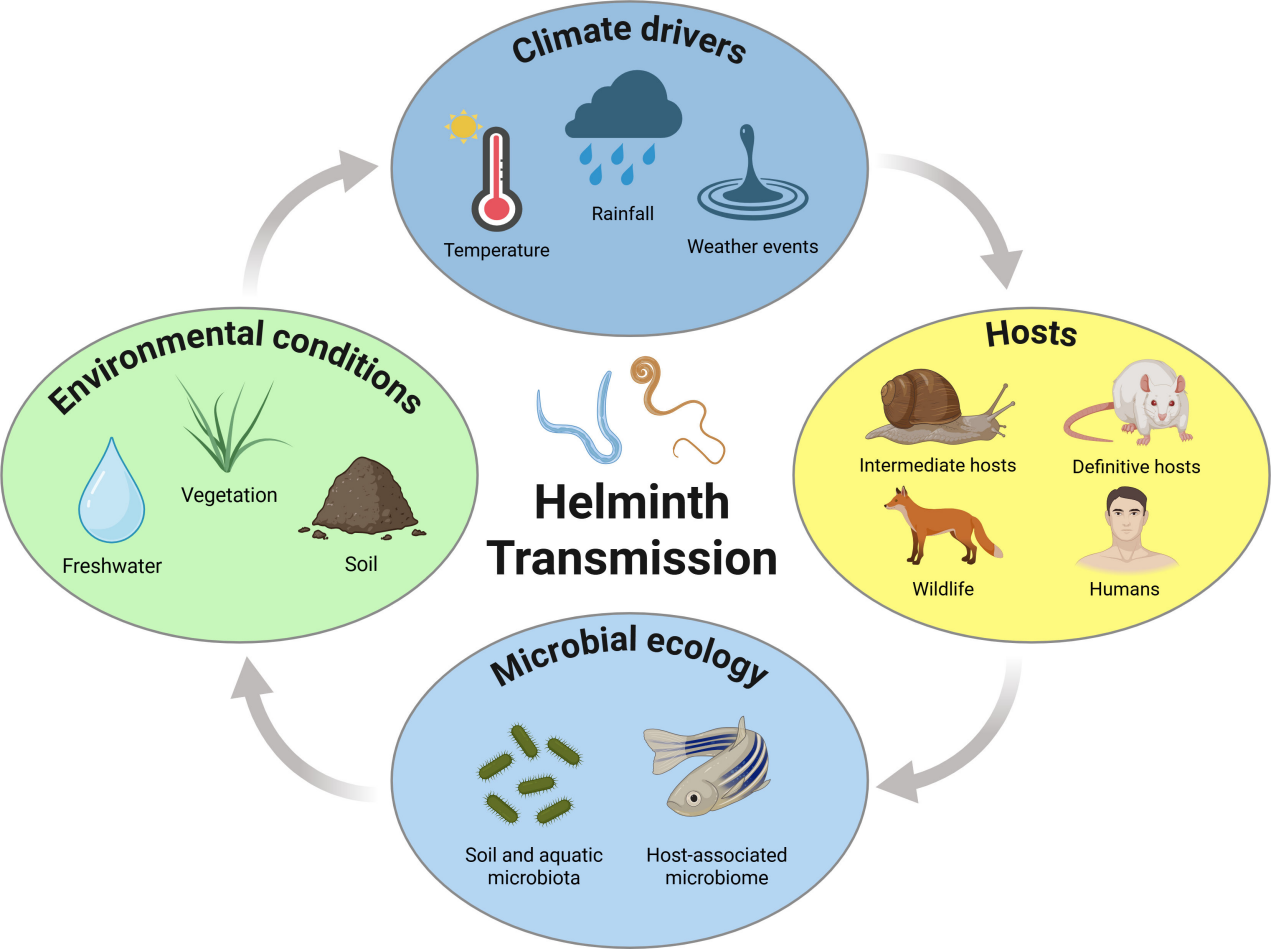
Integrative One Health conceptual model of climate-sensitive helminth transmission. Climate drivers (temperature, rainfall, and extreme weather events), environmental conditions (soil, freshwater, and vegetation), microbial ecology (soil and aquatic microbiota and host-associated microbiomes), and host populations (intermediate and definitive hosts, wildlife, and humans) interact to shape helminth transmission. These domains are interconnected through bidirectional relationships, reflecting the nonlinear and context-dependent nature of transmission dynamics. Anthropogenic environmental change acts as a cross-cutting force influencing all components of the system.

Environmental factors such as water availability, vegetation, and soil structure interact with climatic variables, including temperature and precipitation. Microbial communities influence parasite viability and host susceptibility, while host populations determine opportunities for exposure (15). These interacting drivers can be summarized in an integrative One Health conceptual model illustrating how climate change, environmental conditions, microbial ecology, and host dynamics collectively shape helminth transmission (Fig. 1). Viewed together, these components provide a holistic basis for predicting and monitoring helminth transmission.

## CLIMATE-SENSITIVE MECHANISMS AND ILLUSTRATIVE EXAMPLES

Although helminths vary widely in their ecological requirements, several recurring mechanisms illustrate how climate shapes their biology and transmission across diverse systems.

### Climate effects on parasite biology

Eggs and larvae must often survive in soil, freshwater, marine environments, or on vegetation before infecting a host (16). Experimental evidence shows that temperature directly affects parasite transmission efficiency. For instance, increased temperature enhances the swimming performance and dispersal capacity of *Schistosoma* cercariae, increasing the likelihood of host encounter (14). In aquatic systems, temperature plays a central role in regulating parasite activity and host encounter rates. In contrast, for helminths with environmentally resistant stages in soil, such as soil-transmitted helminths, humidity and soil moisture are key determinants of survival and infectivity, as eggs can remain viable for extended periods under favorable environmental conditions (17, 18).

Heavy rainfall or flooding can disperse infective stages into new habitats, increasing exposure opportunities. In contrast, drought reduces survival but may concentrate hosts around limited water sources, intensifying local transmission (19). These opposing effects illustrate the nonlinear nature of climate–transmission relationships.

### Shifts in intermediate host ecology

Gastropods, insects, crustaceans, and small mammals exhibit climate-sensitive ecological niches. Rising temperatures may expand suitable habitat or extend seasonal windows of host activity (4, 5). Ecological niche modeling studies suggest that climate change may expand the geographic distribution of *Schistosoma japonicum* by increasing habitat suitability for its intermediate host under certain scenarios (20). Hydrological variability alters aquatic habitats essential for many intermediate hosts.

### Changes in definitive host behavior and distribution

Wildlife and domestic animals respond to climate-driven changes in habitat structure, food availability, and migration patterns. Habitat fragmentation and warming climates can increase interactions between humans, livestock, and wildlife, facilitating zoonotic transmission. Domestic animals may become amplifying hosts when environmental suitability enhances parasite survival or intermediate host density (21).

### Emergence in new geographic regions

As temperatures rise, helminths historically restricted to tropical or subtropical climates are increasingly reported in temperate zones (22, 23). Warmer winters and extended warm seasons improve survival of parasite stages and intermediate hosts, enabling establishment in previously unsuitable areas. These shifts challenge historical assumptions about helminth distributions and highlight the need for surveillance systems that incorporate real-time environmental change.

The nematode *Angiostrongylus cantonensis* provides a clear example of climate-sensitive emergence. Its expansion has been linked to temperature-dependent development and the spread of invasive intermediate hosts, with defined thermal thresholds governing larval maturation (24). These findings highlight how species-specific biological parameters can shape responses to environmental change.

### Synergy between climate stress and anthropogenic disturbance

Land-use transformations interact with climatic pressures to intensify transmission (25). Agriculture alters soil and hydrology; urbanization modifies microclimates and biodiversity; and deforestation reshapes wildlife communities. These combined pressures amplify opportunities for parasite persistence and often disproportionately affect regions with limited infrastructure and high vulnerability, reinforcing the importance of integrated One Health strategies.

## NONLINEAR AND CONTEXT-DEPENDENT OUTCOMES

Helminth responses to climate change are inherently heterogeneous and often nonlinear. Rather than producing uniform effects, climatic shifts can generate contrasting outcomes, depending on species-specific biology, ecological thresholds, and local environmental conditions.

Modeling studies indicate that while climate change may expand transmission zones in some regions, it may simultaneously reduce transmission in others where environmental conditions become unsuitable for parasite development or host survival (14). Recent evidence further reinforces this pattern. For instance, climate-based projections for the gastrointestinal nematode *Haemonchus contortus* suggest that transmission potential is likely to decrease across large regions of Africa under future climate scenarios, although localized increases may still occur in specific ecological settings (26). These findings highlight that climatic suitability is constrained by physiological and ecological thresholds, beyond which transmission may decline.

In addition, ecological processes such as biodiversity-driven dilution effects can reduce parasite transmission, demonstrating that environmental change may also decrease infection risk under specific conditions (27). Taken together, these contrasting patterns underscore that helminth transmission cannot be predicted using linear assumptions, but instead emerges from complex, context-dependent interactions across environmental, biological, and ecological domains.

## OPPORTUNITIES FOR PREDICTION, SURVEILLANCE, AND INTERVENTION

Climate-informed modeling and forecasting are essential for anticipating shifts in helminth transmission. Models that incorporate climatic, environmental, and host-related variables can improve predictions of emergence and spatial spread (14, 17). Remote sensing and ecological modeling allow estimation of habitat suitability under future climate scenarios, and when combined with biological data, these approaches can identify regions at risk of intensifying transmission. Improving predictive accuracy will require integration of fine-scale ecological data with long-term environmental monitoring.

### Machine learning and data integration

Machine learning approaches provide new opportunities to synthesize diverse data sets and detect complex interactions that are not apparent through traditional analyses (25). Incorporating helminth-specific biological parameters, such as temperature-dependent developmental thresholds observed in *Angiostrongylus cantonensis*, can further refine predictive models and allow estimation of transmission potential under different climate scenarios (24). These approaches could support the development of dynamic risk maps that are continuously updated based on environmental and biological inputs.

### Environmental and ecological indicators

Monitoring vegetation cover, water quality, soil moisture, and intermediate host abundance provides valuable indicators of transmission potential (12). Microbial assessments may reveal ecological changes that precede shifts in infection patterns, offering early signals of emerging risk. Integrating these indicators into real-time surveillance platforms could enable proactive detection of transmission hotspots before widespread transmission occurs.

### Strengthening One Health surveillance systems

Coordinated surveillance across human, animal, and environmental domains is critical for detecting emerging helminthiases (28). Integrating veterinary and wildlife monitoring with environmental assessments improves early detection of ecological anomalies. Cross-sector data sharing enhances the capacity to identify emerging hotspots and respond promptly. Future systems may benefit from interoperable data platforms that link environmental monitoring, epidemiological surveillance, and genomic or microbiome data.

### Adaptive and climate-informed interventions

Interventions must account for local ecological contexts and variability in climatic conditions. Traditional strategies, including improvements in water and sanitation infrastructure, intermediate host control, and preventive chemotherapy, remain essential. However, climate-informed approaches could enable more adaptive and targeted interventions, such as adjusting treatment timing based on environmental risk thresholds or deploying interventions in response to predicted ecological changes, supporting a transition from reactive to proactive control strategies in the context of climate change.

## CONCLUSION: HELMINTHIASIS IN THE ANTHROPOCENE

Helminths are highly sensitive to environmental and climatic pressures, making them valuable indicators of ecological instability. Their emergence in new regions and shifting transmission dynamics reflect broader transformations associated with the Anthropocene.

Addressing these challenges requires a One Health approach that integrates climatic, environmental, microbial, and host-related determinants. Advances in climate-informed modeling, ecological surveillance, and microbial assessments provide new opportunities to improve understanding and anticipation of changes in transmission dynamics. When combined with coordinated monitoring across human, animal, and environmental sectors, these approaches can support more resilient strategies for prediction and control.

Rather than following linear trajectories, helminthiases emerge from complex and context-dependent interactions across ecological systems. Recognizing helminthiases as dynamic outcomes of climate-driven environmental and microbial processes will be essential for developing predictive, adaptive, and sustainable control strategies in an increasingly climate-unstable world.

## Data Availability

The data that support the findings of this study are available from the corresponding author upon reasonable request.
